# Etanercept in Spinal Cord Injury: A Systematic Review

**DOI:** 10.3390/brainsci16040388

**Published:** 2026-03-31

**Authors:** Lucas Gorial Garmo, Sid Osborn, Emily Hock, Julien Rossignol, Gary L. Dunbar

**Affiliations:** 1 College of Medicine, Central Michigan University, Mount Pleasant, MI 48859, USA; osbor1sh@cmich.edu (S.O.); hock1ea@cmich.edu (E.H.); rossi1j@cmich.edu (J.R.); 2 Program in Neuroscience, Central Michigan University, Mount Pleasant, MI 48859, USA; dunba1g@cmich.edu; 3 Department of Psychology, Central Michigan University, Mount Pleasant, MI 48859, USA

**Keywords:** spinal cord injury, etanercept, tumor necrosis factor-alpha, inflammation, neuroprotection

## Abstract

**Highlights:**

**What are the main findings?**

- In preclinical models of spinal cord injury, TNF-α inhibition with etanercept was associated with reductions in inflammatory markers, tissue damage, and functional deficits in the majority of studies.
- Experimental methodology varied in injury models, dosing strategies, and timing of administration, highlighting significant heterogeneity in the current literature.

**What are the implications of the main findings?**

- Modulation of TNF-α–mediated neuroinflammation represents a promising but not fully elucidated therapeutic strategy in experimental spinal cord injury.
- Standardization of experimental design and further mechanistic investigation are warranted.

**Abstract:**

**Background/Objectives**: Traumatic spinal cord injury (SCI) frequently results in permanent motor and sensory deficits. Tumor necrosis factor-α is rapidly upregulated after SCI and contributes to secondary injury cascades, including microglial activation, cytokine amplification, and blood–spinal cord barrier disruption. Etanercept, a TNF-α inhibitor, has been investigated in modulating post-SCI neuroinflammation. This systematic review synthesizes preclinical evidence evaluating the therapeutic role of etanercept in SCI. **Methods**: A systematic review was conducted in accordance with PRISMA 2020 guidelines. The review was not prospectively registered. PubMed, Scopus, and Web of Science were searched through 9 December 2025. Eligible studies included original investigations of etanercept administered for in vivo mammalian models of SCI. Non-English articles, preprints, conference abstracts, case reports, and reviews were excluded. Risk of bias was assessed independently by at least two reviewers using the SYRCLE tool. Due to heterogeneity in models and dosing strategies, meta-analysis was not performed. **Results**: Of 119 records identified, 36 duplicates were removed. After screening 83 titles and abstracts, 67 were excluded. One additional study was excluded after full-text retrieval. Thus, 15 articles were included. Primary outcomes varied between studies, including inflammation, histopathology, and functional recovery. **Conclusions**: Preclinical evidence suggests that etanercept may attenuate early neuroinflammation after SCI; however, methodological heterogeneity and limited data warrant further investigation. This work was supported by the College of Medicine at Central Michigan University, the John G. Kulhavi Professorship in Neuroscience, and the E. Malcolm Field and Gary Leo Dunbar Endowed Chair in Neuroscience at Central Michigan University.

## 1. Introduction

Spinal cord injury (SCI) remains a major source of global morbidity and mortality. With a global incidence of approximately 574,500 new cases per year, this condition is of significant public health concern [1]. SCI can occur following either traumatic or non-traumatic events involving the spinal cord; however, over 90% of SCI cases are classified as traumatic [2]. Traumatic etiologies include either complete or incomplete injuries. Complete SCI occurs when total severance of neuronal communication at the affected spinal level occurs, while an incomplete SCI refers to partial damage to the affected area. Regardless of type, SCI may result in irreversible damage to nerves essential for communication between the brain and the body, which can lead to permanent sensory and/or motor deficits both at and below the site of injury [3,4].

The injury and recovery processes contributing to the permanent deficits seen following SCI are regarded universally as multi-phasic. The first major stage, denoted as the primary injury phase, results from direct mechanical trauma to the spinal cord, most commonly due to motor vehicle accidents, falls, sports-related injuries, or violence. This primary trauma induces a secondary injury cascade, which involves ischemia, inflammation, and neuronal or cellular death [3,5,6,7,8]. Within this secondary injury cascade, the central nervous system (CNS) uniquely responds with a characteristic healing mechanism, the formation of a glial scar. Although previously considered as being solely inhibitory to functional recovery, the glial scar is now recognized as having both protective and restrictive roles in CNS injury [9,10,11,12,13,14]. Astrocytes responding to inflammation and the resulting immune response drive the formation of the glial scar, creating a protective barrier to limit the spread of inflammation and prevent further CNS damage. However, the glial scar is also described to hinder axonal and neuronal regrowth by imposing both physical and biochemical constraints on peri-lesional spinal cord regions [15,16]. Thus, modulation of glial scar formation, such as by mediating the post-SCI inflammatory responses that promote it, to maximize its benefits while limiting its impedance on post-SCI recovery, remains a key objective of current investigations.

In the immediate phases following SCI, astrocytes and microglia are among the first to activate, initiating an inflammatory cascade that facilitates the recruitment of neutrophils and macrophages to the site of injury. These activated immune cells produce inflammatory mediators, such as various cytokines, proteolytic enzymes, and reactive oxygen species (ROS), which can contribute to further damage. In particular, the tumor necrosis factor (TNF) family of cytokines has been extensively investigated following SCI, as it is known to play a key role in promoting inflammation, and excessive production of TNF has been linked to varied CNS inflammatory pathologies [17]. TNF can bind to both tumor necrosis factor receptor-1 (TNFR-1) and receptor-2 (TNFR-2). However, TNFR-1 is suggested to contain a death domain associated with apoptosis and neurodegeneration, whereas TNFR-2 appears to be described as having neuroprotective properties [18]. Furthermore, TNF is known to induce the expression of other cytokines and chemokines, thus playing a key role in post-SCI inflammatory processes, inspiring investigation into the therapeutic potential of modulating the production and/or relative activities of TNF and its receptors. Using PCR, Pan et al. (2002) [19] conducted a study demonstrating that TNF-α mRNA levels in the spinal cord increase as early as 15 min post-SCI, with peak expression observed at 1 h post-injury. This early and transient surge of TNF-α suggests its crucial role in initiating the secondary inflammatory response, triggering the production of other pro-inflammatory cytokines (e.g., IL-1β, IL-6) and chemokines (e.g., CCL2, CXCL10), which contribute to immune cell recruitment and secondary damage. Although TNF has been implicated in neuronal and oligodendrocyte death post-SCI, and elevated TNF demonstrates increased apoptosis in an injured spinal cord within the early stages following injury, increased TNF expression 7 days after SCI has been shown to improve tissue healing [18]. Thus, it can be surmised that TNF demonstrates a multi-phasic and multi-faceted role in the post-SCI microenvironment, as increased TNF levels have been suggested to be deleterious in the acute phases but have also exhibited neuroprotective effects chronically. Therefore, the temporality of TNF release is an important factor to consider in post-SCI recovery.

To date, there is no universally accepted standardized pharmaceutical intervention in the management of SCI. Current clinical management of SCI emphasizes rapid diagnosis and neuroprotective interventions within the acute injury phase, with surgical decompression of the spinal cord to maintain adequate spinal cord perfusion being highlighted as a priority if deemed feasible [5,7]. High-dose methylprednisolone sodium succinate (MPSS) has been previously identified as having potential therapeutic benefits, namely by inhibiting lipid peroxidation, enhancing spinal cord perfusion, and limiting the inflammatory response; however, a joint statement released by the American Association of Neurological Surgeons and the Congress of Neurological Surgeons was later published that discouraged the use of MPSS, citing an increased risk of wound infections, gastrointestinal hemorrhage, sepsis, pulmonary embolism, pneumonia, and death alongside its use after SCI [20].

Etanercept (ETN), a biologic TNF inhibitor, has recently gained special attention within the SCI discourse [21]. Clinically, ETN is used primarily in the management of rheumatologic conditions, such as rheumatoid arthritis, to limit arthritic damage and regain functional recovery. However, the use of ETN has also been indicated for use in other immune-mediated conditions, such as psoriasis or ankylosing spondylitis [22]. Although ETN belongs to a broader class of TNF inhibitors, which includes infliximab and adalimumab, these are monoclonal antibodies that directly target TNF-α and carry a certain risk of immunogenicity and subsequent anti-drug antibody formation, which can hinder treatment efficacy. Conversely, ETN uniquely acts as a soluble TNF receptor fusion protein, binding to both TNF-α and TNF-β to effectively block their effects and facilitate their removal from the systemic circulation. This mechanistic difference is believed to contribute to a lower risk of immunogenicity and certain infections, particularly granulomatous infections, with ETN use compared to monoclonal anti-TNF therapies, such as infliximab [23,24].

Despite numerous clinical studies demonstrating the dramatic alleviation of symptoms with the administration of ETN in various disk-related pathologies—such as sciatica, radiculopathy, and myelopathy—clinical investigation of ETN in SCI remains scarce [21,25,26,27,28,29]. Since TNF expression is known to increase substantially after SCI and results in several downstream inflammatory effects, ETN offers a feasible opportunity for therapeutic intervention and modulation of the secondary injury cascade. The current review sought to synthesize and describe the current literature relating to the therapeutic potential of ETN in SCI.

## 2. Materials and Methods

### 2.1. Study Design

The authors performed a systematic review of the literature in accordance with the Preferred Reporting Items for Systematic Reviews and Meta-Analyses (PRISMA) 2020 guidelines [30]. As this search was originally initiated as a preclinical evidence synthesis and not a prospectively planned systematic review, this review or its protocol was not prospectively registered. However, the eligibility criteria, outcome definitions, and methodology were prespecified prior to data extraction, and the search was re-conducted later to ensure compliance with the PRISMA guidelines. Included were studies accessible as of 9 December 2025, and reported on the use of ETN in the management of SCI. The studies must have involved an in vivo mammalian example of SCI pathology (e.g., mouse, rat), used ETN as either an experimental or control treatment, and must have been originally published in the English language. Pre-print articles, case reports, conference abstracts, and other reviews were among the types of publications excluded. Table 1 describes the full inclusion and exclusion criteria.

### 2.2. Study Selection

The search terms utilized in the queries of three databases (PubMed, Scopus, and Web of Science) were based on the Medical Subject Headings (MeSH) trees of “Etanercept” and “Spinal Cord Injuries”. When possible, MeSH terms were expanded and converted for equivalency for use in Scopus and Web of Science. The strategy employed Boolean operators to combine terms related to the intervention (‘Etanercept’, ‘TNFR-Fc Fusion Protein’) and the condition (‘Spinal Cord Injury*’). In PubMed, both MeSH terms and Title/Abstract tags ([tiab]) were utilized to ensure comprehensive coverage. No database filters for language or study type were applied during the initial search phase. At the time of searching, gray literature (e.g., conference abstracts, unpublished dissertation works) was not specifically targeted in the search strategy and was subsequently excluded during the screening phases, in accordance with the prespecified inclusion and exclusion criteria (Table 1). Manual reference screening of included articles was not performed. The final search queries utilized in this review are provided as supplementary material (File S1). The searches were initially conducted by one reviewer on 14 August 2024. The search results were compiled and exported to Zotero 6.0 (Corporation for Digital Scholarship, Vienna, VA, USA) for de-duplication, and a blinded review was conducted using the Rayyan web application. The search was re-conducted on 9 December 2025, in which 3 new articles were identified and included for screening.

Initial screening of article titles and abstracts within Rayyan involved the assignment of “include”, “exclude”, or “maybe” labels to each article by at least two of three reviewers, each of whom was blinded to the votes of the others. Using the labeling feature of Rayyan, each article receiving either “exclude” or “maybe” was also assigned a reason for potential exclusion. After each article was screened, reviewers were unblinded and met to resolve any conflicts and vote on the inclusion or exclusion of articles tagged as “maybe”. Subsequently, blinded review was re-enabled, and the remaining articles were retrieved and uploaded to Rayyan for full-text review. During full-text review, each of the articles was reviewed independently by each of the three reviewers, and was assigned “include”, “exclude”, or “maybe”. After the full-text review was completed, reviewers were unblinded and once again met to resolve any conflicts or uncertainties. The remaining articles were then divided evenly amongst reviewers for manual data abstraction and synthesis into a shared spreadsheet. At the conclusion of data abstraction, reviewers reconvened to validate abstracted information and resolve any uncertainties.

### 2.3. Abstracted Data

The data abstracted from each article included the type of SCI (e.g., contusion, compression, etc.), the model demographics (e.g., species, sex, weights), SCI region (e.g., cervical, thoracic, lumbar), study outcomes and timepoints, modality of ETN administration, and dosage and frequency of ETN. A consolidated depiction of abstracted article data can be visualized in Table 2. Studies were grouped for synthesis based on intervention type, injury model, timing of administration, and outcome type, shown in Table 3. Due to the small sample of included studies and heterogeneity in animal model, type of SCI, and timing/route of ETN administration, findings were synthesized narratively. No formal sensitivity analyses were conducted. Overall confidence in outcomes was interpreted descriptively in the context of study-level risk-of-bias assessments. Risk of bias was independently assessed by two reviewers using the SYRCLE (Systematic Review Centre for Laboratory Animal Experimentation) risk-of-bias tool, which is specifically adapted for preclinical animal studies [31]. The SYRCLE tool allows reviewers to evaluate ten domains across selection, performance, detection, attrition, reporting, and other potential sources of bias in interpreting a specified outcome of interest. In the current review, the SYRCLE tool was utilized to identify potential sources of bias that might affect the interpretation of etanercept’s effectiveness in experimental SCI. Prespecified critical domains for determining overall study-level risk of bias were: (1) random sequence generation, (2) blinding of outcome assessment, and (3) incomplete outcome data. Allocation concealment was evaluated but not considered a primary determinant of overall classification due to limited reporting across included studies. It is important to note that risk of bias assessment was based on reported methodological details, and that given evolving reporting standards, absence of explicit reporting of certain factors should not be interpreted as absence of methodological rigor. Overall risk of bias was classified as low (all critical domains rated low risk), moderate (at least one critical domain rated unclear, none rated high), or high (at least one critical domain rated high risk). No numerical scoring or summation across domains was performed, in accordance with SYRCLE guidance. In the case of a disagreement in overall classification, a third independent reviewer was consulted.

## 3. Results

### 3.1. Search Results

**Figure 1:**
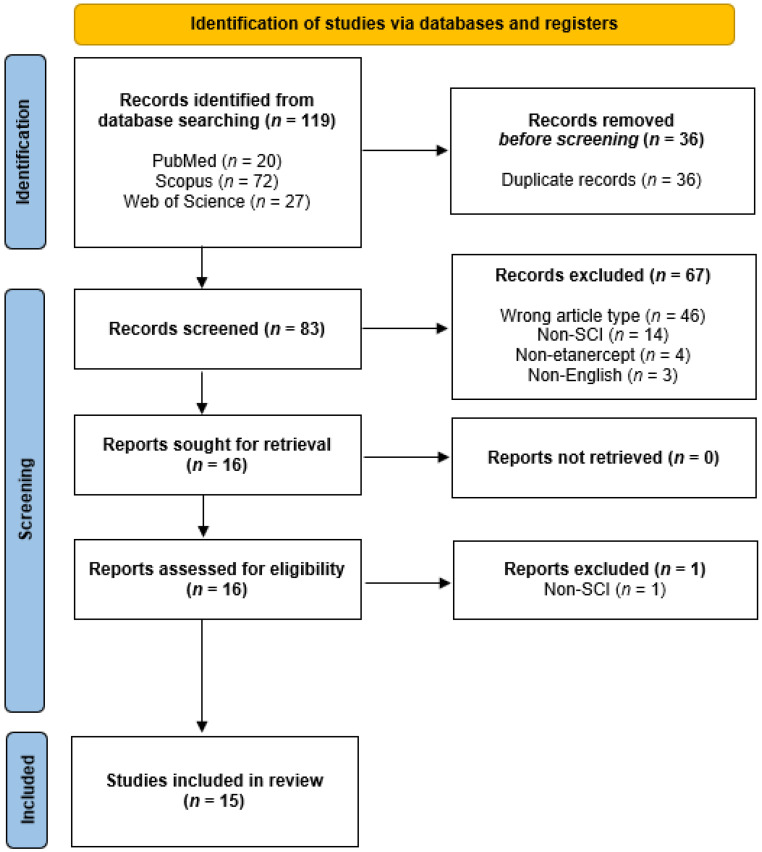
PRISMA flowchart depicting the study identification process.

The study identification process can be visualized in the PRISMA flowchart (Figure 1). One-hundred-sixteen citations were originally identified from the query of three databases (PubMed, Scopus, Web of Science), with three additional articles being identified upon subsequently re-conducting the search *(n* = 119). After de-duplication within Zotero, 36 records were removed, and the remaining 83 were uploaded to Rayyan for blinded review of titles and abstracts. This initial screening phase resulted in the exclusion of 67 records for not meeting the inclusion/exclusion criteria (Table 1). The remaining 16 articles were sought for full-text review, after which one article was subsequently excluded for investigating spinal nerve ligation rather than spinal cord injury [47]. Thus, 15 studies in total were included in the current review.

### 3.2. Effect of ETN on Inflammatory Cytokines and Oxidative Stress

Across the included studies, ETN consistently demonstrated a modulatory effect on pro-inflammatory cytokines following SCI, particularly TNF-α and IL-1β [32,33,34,35,36,37,40,41,42,44,45,46]. Twelve studies evaluated inflammatory or oxidative stress-related outcomes, all of which reported improvement in at least one measure.

Reductions in TNF-α were observed across multiple experimental paradigms and time points. For instance, ETN reduced TNF-α levels within hours of injury (1–6 h) and maintained suppression into later time points (24–48 h) in both rat and transgenic TNF-α models [35,44]. At the receptor level, ETN was associated with decreased TNFR-1 and TNFR-2 expression as early as 6–12 h post-injury, along with reduced cytokine expression in both neuronal and glial populations [36]. Additional inflammatory markers, including IL-1β and IL-6, were similarly reduced across studies, and ETN also suppressed microglial activation, as evidenced by decreased OX-42 (CD11b) expression at 14 and 28 days post-injury with early treatment [32,33,34,44,45,46].

ETN also demonstrated consistent effects on oxidative stress pathways. Reductions in nitrotyrosine and neutrophil infiltration were reported in early preclinical models [32,33], while other studies demonstrated broader reductions in oxidative stress markers alongside increased antioxidant enzyme activity [44]. These effects were observed across multiple delivery strategies, including systemic administration and nanoparticle-mediated delivery systems, the latter of which further demonstrated downregulation of NF-κB pathway activation and pro-inflammatory transcriptional activity [45,46].

Timing of administration emerged as a critical determinant of anti-inflammatory efficacy. Early ETN delivery—within hours of injury or immediately post-injury—resulted in sustained reductions in cytokine expression and microglial activation [34,38,44]. In contrast, delayed administration (e.g., initiation at 14 days post-injury) failed to significantly reduce inflammatory markers [34], and in some models, delayed treatment was associated with less favorable inflammatory profiles [39].

Combination therapies frequently enhanced the anti-inflammatory effects of ETN. For example, ETN combined with erythropoietin produced greater reductions in TNF-α than ETN alone, although erythropoietin monotherapy remained more effective in some comparisons [42]. Similarly, the combination with salvianolic acid B further reduced TNF-α mRNA expression compared to ETN alone [37]. In contrast, ETN monotherapy was not effective in all studies; one study demonstrated that ETN monotherapy failed to significantly reduce inflammatory markers compared with its combination with dexamethasone [33].

Notably, differences were observed between systemic and central delivery strategies. Systemic ETN administration consistently reduced TNF-α and other pro-inflammatory cytokines across multiple studies and time points [32,33,34,35,36,37,40,41,42,44,45,46]. In contrast, central delivery demonstrated more limited and context-dependent effects, with evidence of reduced microglial activation following early intrathecal administration, but no significant benefit when treatment was delayed [34]. Additionally, epidural ETN did not demonstrate significant anti-inflammatory or downstream functional benefit in one study, suggesting that the route of delivery may influence the magnitude and consistency of cytokine modulation [41].

### 3.3. Effect of ETN on Histopathological Outcomes and Neuroprotection

Eleven studies evaluated histopathological or neuroprotective outcomes following ETN treatment, with nine of these studies reporting improvement in at least one measure. Overall, ETN was associated with reduced structural damage and improved preservation of spinal cord architecture after SCI [32,35,36,40,43,44,45,46].

Reductions in lesion severity were reflected by decreased cystic cavity formation, smaller lesion areas, and increased residual tissue at the injury epicenter [36,40]. ETN treatment also resulted in greater sparing of both gray and white matter, with attenuation of myelin loss in dorsal and lateral columns observed in early studies [32,36]. These structural improvements were corroborated by microscopy findings demonstrating reduced axonal swelling, decreased necrosis, and improved ultrastructural integrity [43,44].

Treatments with ETN also exerted significant anti-apoptotic effects. Multiple studies reported reductions in TUNEL-positive cells and decreased caspase activity at early and subacute time points (e.g., 6 h to 7 days post-injury), indicating suppression of programmed cell death pathways [36,40].

In combinatorial regimens, ETN enhanced the survival and integration of transplanted neural stem cells. Combination therapy with ETN and neural stem cells resulted in reduced apoptosis, improved axonal myelination, and smaller lesion cavities compared to stem cell therapy alone [40]. Similarly, nanoparticle-mediated delivery systems were associated with increased survival of motor neurons and reduced injury area compared to controls [45,46]. However, these benefits were not universal. Some studies reported no significant reduction in lesion size or injury severity, particularly at early time points (24–72 h) or in specific treatment paradigms [41,42]. Additionally, in one study, ETN did not significantly reduce lesion size at 8 weeks post-injury, suggesting that long-term histopathological benefits may be limited under certain conditions [41].

Differences in histopathological outcomes were also observed between delivery strategies. Systemic ETN was consistently associated with reduced lesion size, decreased apoptosis, and improved tissue preservation across multiple models [32,35,36,40,43,44,45,46]. In contrast, central delivery approaches demonstrated less consistent structural benefit, with no significant reduction in lesion size observed following epidural ETN administration in one study [41]. These findings suggest that systemic delivery may confer broader neuroprotective effects, whereas central delivery may have a more limited impact on structural outcomes.

### 3.4. Effects of ETN on Functional and Behavioral Recovery

Functional outcomes were assessed in most of the included studies, with most reporting improvement in at least one measure following ETN treatment. ETN was associated with enhanced locomotor recovery, improved coordination, and improved electrophysiological outcomes, including somatosensory evoked potentials [32,36,37,38,40,42,43,44].

Improvements in Basso, Beattie, and Bresnahan (BBB) locomotor scores were among the most consistently reported findings, particularly in studies utilizing early systemic administration. For example, early intramuscular ETN administration (2–4 h post-injury) resulted in greater improvements in motor function and electrophysiological recovery compared to delayed administration (12–24 h) [38]. Similarly, intraperitoneal ETN improved locomotor function beginning at approximately 2 weeks post-injury and was associated with increased neuronal and oligodendrocyte survival [36].

Functional recovery was strongly influenced by the timing of treatment. Early administration consistently resulted in improved locomotor outcomes, whereas delayed administration—particularly in the chronic phase (e.g., 14 days post-injury)—failed to improve coordination, strength, or overall functional performance [34,39]. In some models, delayed ETN treatment was associated with worsened tissue outcomes, further emphasizing a narrow therapeutic window [35].

Combination therapies also demonstrated superior functional outcomes. ETN combined with erythropoietin resulted in greater BBB scores compared to ETN alone, although erythropoietin monotherapy remained more effective in some comparisons [42]. Similarly, ETN combined with neural stem cell transplantation improved motor recovery compared to neural stem cell transplantation alone [40]. Combination with salvianolic acid B also enhanced functional recovery beyond ETN monotherapy [37].

Central delivery approaches demonstrated selective benefits. Early intrathecal ETN reduced neuropathic pain behaviors, including mechanical allodynia, and decreased microglial activation, whereas delayed administration had no significant effect [34]. However, not all delivery strategies were effective; epidural ETN did not improve functional recovery or lesion size in one study, whereas an alternative TNF inhibitor (XPro1595) demonstrated benefit when delivered via the same route [41].

Despite generally positive findings, two studies reported no significant functional improvement with ETN treatment, particularly in delayed systemic administration paradigms or when compared to alternative TNF-targeting strategies [39,41].

Systemic ETN was associated with improvements in locomotor recovery and electrophysiological outcomes in the majority of studies, particularly when administered early after injury [32,36,37,38,40,42,43,44]. In contrast, central delivery demonstrated more selective effects, with early intrathecal administration improving neuropathic pain behaviors but not consistently enhancing overall locomotor recovery [34]. Additionally, epidural ETN failed to improve functional outcomes in one comparative study, whereas an alternative TNF inhibitor delivered via the same route was effective [41]. Together, these findings suggest that systemic delivery may be more reliable for motor recovery, while central delivery may preferentially target localized inflammatory or pain-related processes.

### 3.5. Risk of Bias Assessment

Risk of bias was assessed using the SYRCLE tool across ten methodological domains. Overall classification was based on prespecified primary critical domains: random sequence generation, blinding of outcome assessment, and completeness of outcome data (Table 4). Allocation concealment was not reported in any study. Given this uniform absence of reporting, this domain was evaluated but not weighted as a primary determinant of overall classification, as including it would not allow for meaningful discrimination of relative risk of bias between studies. Furthermore, as reporting standards in preclinical research are continuously evolving, an absence in reporting of allocation concealment does not necessarily equate to a lack of rigor. Random sequence generation was explicitly reported in 9 of 15 studies (60%), while 6 studies (40%) did not clearly describe random allocation procedures. Baseline characteristics were adequately described and comparable across groups in all included studies. Blinding of outcome assessment was explicitly reported in 11 of 15 studies (73%), most commonly for behavioral scoring and/or histopathologic evaluation. Four studies did not clearly state whether outcome assessors were blinded. Incomplete outcome data were low risk across all studies, with no evidence of unexplained attrition or selective exclusion of animals. Selective outcome reporting was not identified in any included study. Other potential sources of bias were generally low risk; however, several studies noted small group sizes or limited reporting transparency. Using the prespecified three primary critical domains framework, 8 studies (53%) were classified as low risk of bias, having satisfied all primary domains. The remaining 7 studies (47%) were classified as moderate risk of bias due to at least one unclear primary domain. No studies were judged to be at high risk of bias. Overall, the absence of studies classified as high-risk, coupled with heterogeneity in the reporting of randomization procedures and small sample sizes, suggests that although reported effects of etanercept across studies are generally consistent, confidence in these conclusions remains limited.

## 4. Discussion

Despite our growing understanding of SCI pathophysiology, treatments for the ensuing sensory and motor deficits remain scarce. Post-injury inflammatory cascades lead to the eventual formation of a glial scar, which may prevent axonal and neuronal regrowth; therefore, modulation of these cascades is a key area of research interest. Although the TNF family of cytokines, which play a major role in inciting the secondary injury cascade, have been extensively studied, the clinical potential of the TNF inhibitor, ETN, in SCI remains to be fully elucidated. This systematic review highlights and synthesizes the existing in vivo evidence for the therapeutic potential of ETN in SCI. Across the literature, ETN consistently attenuates inflammatory responses, decreases expression of proinflammatory cytokines, preserves neural and glial components, and demonstrates improvements in histopathological and functional outcomes compared with control conditions. Collectively, these findings reiterate the role of TNF-α as a key mediator of the secondary injury cascade following SCI and warrant further continued exploration of TNF inhibition as a potential therapeutic strategy within the acute and subacute phases of SCI.

As a TNF inhibitor, ETN belongs to a broader pharmacological class that includes several clinically approved agents, each with distinct mechanisms of action and safety profiles. Infliximab and adalimumab are monoclonal antibodies that directly neutralize TNF-α, while ETN is unique in functioning as a soluble decoy receptor, specifically a recombinant fusion protein consisting of two TNFR-2 extracellular domains linked to the Fc region of IgG1, that competitively binds both TNF-α and TNF-β, thereby preventing their interaction with cell-surface receptors [23]. This mechanistic distinction confers important differences: unlike monoclonal anti-TNF antibodies, ETN does not trigger complement fixation or induce complement-dependent cytotoxicity, and carries a comparatively lower risk of immunogenicity, anti-drug antibody formation, and granulomatous infections, including reactivation of latent tuberculosis, relative to other anti-TNF agents [23,24]. These properties may be particularly relevant in the SCI context, where the patient population is already at elevated infection risk.

### 4.1. Role of TNF Modulation in SCI

This review marks an important shift in the evaluation of SCI treatment. By focusing on the role of ETN in post-injury inflammatory processes, findings of the current review suggest that post-injury elevations of TNF-α are associated with amplification of the secondary injury cascade, leading to neuronal apoptosis, oxidative stress, and lesion expansion. The secondary injury cascade represents a complex and temporally dynamic series of cellular and molecular events that amplify the damage initiated by primary mechanical trauma. Immediately after injury, disruption of the blood-spinal cord barrier (BSCB) permits infiltration of peripheral immune cells, while resident microglia and astrocytes rapidly activate in response to damage-associated signals [3,5,6,7,8]. This neuroinflammatory response drives the production of ROS, pro-inflammatory cytokines, and proteolytic enzymes that contribute to progressive tissue destruction. The centrality of these processes is reflected across the included studies: Genovese et al. (2006) documented significant post-SCI upregulation of iNOS, nitrotyrosine, COX-2, TNF-α, and IL-1β, alongside elevated MPO activity reflecting neutrophil infiltration, in untreated injured animals [32]. Oxidative stress is further evidenced by the significant reductions in antioxidant enzyme activity, including superoxide dismutase (SOD) and catalase (CAT), and elevations in malondialdehyde (MDA) and adenosine deaminase (ADA) observed in injured controls by Hasturk et al. (2018) at 1, 6, and 24 h post-SCI [44].

Apoptosis represents another major mechanism of secondary neuronal and oligodendroglial loss: Chen et al. (2011) demonstrated significant upregulation of TNFR-1, TNFR-2, caspase-3, and caspase-8, with TUNEL-positive neurons and oligodendroglia detectable from 12 h through 1 week post-injury in untreated controls [36]. A pro-apoptotic shift in Bcl-2 family signaling—including Bax and FasL upregulation with reciprocal Bcl-2 reduction, was similarly documented in injured untreated animals by Genovese et al. (2007) [33]. Wang et al. (2014) further demonstrated that post-SCI TNF-α directly activates caspase-mediated apoptotic pathways in neural cells within the first 1–3 days following injury, providing a mechanistic link between cytokine upregulation and cellular loss [40]. Together, these processes, cytokine amplification, oxidative stress, neutrophil infiltration, and apoptotic signaling, constitute the core of the secondary injury cascade and form the mechanistic basis for therapeutic targeting of TNF-α in SCI.

Within the broader neuroinflammatory landscape of SCI, TNF-α occupies a particularly central role as an upstream orchestrator of multiple downstream injury processes, providing a compelling rationale for its therapeutic targeting. Evidence from the included studies illustrates that TNF-α modulation may represent a convergence point at which numerous secondary injury mechanisms can be affected. Genovese et al. (2006) demonstrated that ETN-mediated TNF-α suppression produced downstream reductions in IL-1β, iNOS, nitrotyrosine, COX-2, and MPO activity, indicating that blockade of TNF-α propagates broadly into the inflammatory cascade [32]. This pattern was corroborated by a follow-up study by Genovese et al. (2007), in which combined ETN and dexamethasone treatment reduced both cytokine expression and apoptotic markers simultaneously; however, this benefit was not seen for either ETN or dexamethasone monotherapies [33]. The dose-dependent relationship between TNF-α activity and tissue damage was demonstrated by Chi et al. (2010), who showed that suppression of TNF-α prior to injury significantly reduced spinal cord lesion size in transgenic rats, while amplification of TNF-α activity through delayed ETN resulted in significantly greater lesion size [35]. More recently, Sun et al. (2019) and Shen et al. (2021) demonstrated that ETN-mediated TNF-α neutralization extended to macrophage phenotype regulation, shifting polarization from pro-inflammatory M1 toward anti-inflammatory M2 profiles, with corresponding reductions in IFN-γ and increases in TGF-β [45,46]. As TNF-α exerts its effects through two distinct receptor subtypes: TNFR-1, which contains a death domain associated with apoptosis and neurodegeneration, and TNFR-2, which has been associated with neuroprotective signaling [18,20], the temporally variable and mechanistically opposed roles of these receptor subtypes underscore why the timing of TNF-α modulation is critical, and why non-selective blockade of TNF-α by ETN must be considered in light of both its pro-inflammatory and potentially neuroprotective downstream effects.

SCI is associated with a spectrum of neuropathic sequelae that extends beyond motor deficits, including central neuropathic pain, allodynia, spasticity, and electrophysiological conduction deficits, each of which reflects distinct aspects of post-injury neural dysfunction. The included studies addressed several of these phenotypes. With respect to pain and sensory outcomes, Marchand et al. (2009) [34] specifically investigated mechanical allodynia following thoracic hemisection in Wistar rats using von Frey filament testing, demonstrating that immediate intrathecal ETN, initiated 30 min post-injury and delivered at 50 µg/rat/day via osmotic pump for 7 days, significantly reduced mechanical allodynia at 1, 2, 3, and 4 weeks post-SCI. Critically, delayed ETN administration, initiated 14 days post-injury, had no significant effect on allodynia, underscoring the role of intervention timing for this outcome domain [34]. Novrup et al. (2014) also assessed thermal hyperalgesia and found no significant effect of either systemic or central ETN on this measure [41]. Locomotor deficits were the most commonly evaluated phenotype across the included studies, with early-administered ETN predominantly producing significant improvements across these measures. Electrophysiological deficits were evaluated in two studies: Bayrakli et al. (2012) found significantly better somatosensory evoked potential recovery in ETN-treated rabbits at 2 weeks post-SCI compared to untreated controls [38], and Sun et al. (2019) demonstrated significantly greater motor-evoked potential amplitudes in the ETN-nanoflower group at 8 weeks [45].

In the preclinical studies included in the current review, ETN’s action of blocking TNF appears to reduce proinflammatory signaling and mitigate tissue damage. Many studies report decreased microglial activation and bolstered neuronal survival, suggesting ETN may also confer neuroprotective effects beyond anti-inflammatory mechanisms. Importantly, these biologic effects may translate into the improved functional outcomes seen in the majority of included studies, such as improved locomotive recovery post-injury.

### 4.2. Systemic Versus Central Administration

A fundamental consideration in the translational development of etanercept (ETN) for spinal cord injury (SCI) is its limited ability to cross an intact BSCB. As a large recombinant fusion protein, ETN does not readily penetrate the BSCB under normal physiological conditions [22,39]. Despite this theoretical limitation, systemic administration was associated with positive central nervous system effects in most of the included studies, including reductions in spinal cord TNF-α and IL-1β levels, suppression of apoptotic signaling, and improvements in histopathological and functional outcomes.

Two primary mechanisms may explain this apparent paradox. First, the secondary injury cascade following SCI disrupts BSCB integrity, particularly in the acute phase, which may permit transient penetration of systemically administered agents into the injured spinal cord [3,5]. This is supported by findings of reduced perilesional edema and increased white matter sparing in ETN-treated animals [32,33]. Second, peripheral neutralization of circulating TNF-α may attenuate the systemic inflammatory milieu, thereby indirectly reducing central neuroinflammation. This mechanism is supported by Wang et al. (2014), who demonstrated that early intraperitoneal ETN reduced TNF-α expression and caspase-mediated apoptosis within the injured cord [40].

In contrast, central delivery strategies reported in the two reviewed studies were not entirely uniform. Immediate intrathecal ETN administration reduced mechanical allodynia and microglial activation, whereas delayed administration was ineffective, underscoring the importance of timing [34]. Similarly, epidural ETN delivery did not improve functional recovery or reduce lesion size in one study, in contrast to the selective soluble TNF inhibitor XPro1595 delivered via the same route [41]. These findings suggest that central delivery alone may not improve efficacy and raise the possibility that ETN’s non-selective inhibition of both soluble and transmembrane TNF may influence outcomes, depending on route and timing of administration.

Recent studies have attempted to address BSCB penetration limitations through targeted delivery systems. Sun et al. (2019) demonstrated that intravenously administered ETN-loaded nanosheets produced significant improvements in macrophage polarization, lesion size, locomotor recovery, and electrophysiological outcomes [45]. Similarly, Shen et al. (2021) reported that ETN-loaded nanoparticles enhanced functional recovery, neuronal sparing, and M1-to-M2 macrophage polarization compared to free ETN, without evidence of systemic toxicity [46]. These findings suggest that nanoparticle-mediated delivery may represent a promising strategy to overcome BSCB-related limitations and enhance the therapeutic potential of ETN in SCI.

The included studies primarily consisted of short-duration preclinical experiments and, in consistency with this design, did not formally assess adverse effects classically associated with ETN. One study mentioned rates of surgical site infection in two animals, one of which received erythropoietin and the other triple-combinatorial treatment of erythropoietin, etomidate, and ETN [42]. Aside from investigations into ETN encapsulation in nanoparticles [45,46], assessments of toxicity were not conducted by the studies included in this review. Shen et al. (2021), using ETN-loaded nanoparticles, specifically conducted a systemic cytotoxicity assessment via hematoxylin-eosin staining of major organs at days 1, 7, and 28 post-SCI and reported no significant systemic organ toxicity attributable to the treatment [46]. Sun et al. (2019), using ETN-loaded nanoflower constructs, similarly assessed major organs histologically at 8 weeks and found no evidence of systemic toxicity [45]. These are the only two included studies to formally evaluate systemic safety, and both employed nanoparticle delivery vehicles rather than free ETN. One finding of safety relevance comes from Chi et al. (2010), who demonstrated that delayed post-injury ETN (administered 4 days after SCI in TNF-α transgenic rats) resulted in significantly increased spinal cord lesion size compared to untreated controls, in contrast to the significantly reduced lesion size observed with pre-treatment [35]. Although not a classical adverse effect, this finding underscores that delayed or mistimed ETN administration may be detrimental, a critical safety consideration for any translational application.

Nevertheless, ETN has a well-documented clinical adverse effect profile, including an elevated risk of serious systemic infections and the reactivation of latent tuberculosis [23,24]. These risks are particularly salient for the SCI population, who already face increased susceptibility to infection following CNS trauma. While ETN’s systemic infection risk is generally lower than that of other anti-TNF agents (e.g., infliximab), which may be a favorable consideration for SCI patients, the degree of immunosuppression it confers in the acute phases following SCI has not been directly evaluated and warrants attention in future translational investigations.

Among the included studies that investigated systemic delivery, doses of ETN tended to range from 1.25 mg/kg to 5 mg/kg, with one study utilizing a dose of 10 mg/kg and another using a fixed dose of 125 µg/mouse; for central delivery, dosing was considerably lower (Table 2). In comparison, the standard clinical dosing regimen of ETN is generally 50 mg administered subcutaneously once weekly for rheumatoid arthritis, with doses of 50 mg twice weekly having been proven effective in improving psoriasis and psoriatic arthritis [22].

Given its systemic side effect profile and restricted BSCB penetration, localized ETN may be theorized to be a good candidate for investigation within acute SCI. Despite this, only two studies included in this review explored the central administration of ETN, as systemic delivery modalities represented the predominant route of administration, as well as accounted for most positive findings. Across studies, systemic ETN suppressed TNF-α and IL-1β expression, reduced oxidative stress and apoptosis, and improved both histological integrity and locomotor recovery. However, the limited number of studies employing central delivery mechanisms of ETN may contribute to the observed heterogeneity in results, limiting a definitive comparison. Direct comparisons of systemic and central administration of ETN within SCI remain sparse and further exploration is warranted to determine optimized dosing and delivery strategies.

### 4.3. Temporal Dependency

Notably, the timing of treatment post-SCI appeared to impact its therapeutic efficacy. In studies that explored temporality, earlier initiation of ETN post-SCI—particularly within hours of injury—enhanced clinical and electrophysiological recovery as compared with later timepoints. Similarly, some studies indicated that maximal biochemical and histopathologic benefits were observed with immediate or 6 h dosing, with diminished efficacy seen by 24 h. Pre-treatment with ETN also seemed to significantly reduce TNF-α expression and spinal cord lesion size compared with treatments started days after injury. Although conclusions should be interpreted with caution due to the scarcity of available data, findings thus far suggest that prolonged or late TNF blockade by ETN may offer diminished efficacy in experimental SCI than earlier treatment regimens. Moreover, the immediate administration of agents such as ETN presents significant translational challenges in clinical settings of acute, traumatic SCI, so a wider therapeutic window must be established before translational consideration. Therefore, further investigation regarding the temporal dependency of ETN’s effects after SCI is warranted.

### 4.4. Role of ETN in Other Neurologic Injuries

Although the primary focus of this review was on spinal cord injury, ETN has also been investigated in other central nervous system injuries, most notably traumatic brain injury (TBI). TNF-α is rapidly elevated following TBI and contributes to secondary neuroinflammatory cascades that share mechanistic parallels with those observed in SCI, including microglial activation, cytokine amplification, and progressive neuronal loss. Preclinical investigations have demonstrated that systemic administration of ETN attenuates early microglial TNF-α expression and improves neurological outcomes in rat models of TBI [48]. These findings are consistent with the preclinical SCI evidence synthesized in the current review, wherein early ETN-mediated suppression of TNF-α and downstream neuroinflammation was associated with improved histopathological and functional outcomes. At the clinical level, observational data from a large series of patients with chronic neurological dysfunction following brain injury or stroke suggest that perispinal etanercept administration may produce rapid and sustained neurological improvements in some individuals [49], though the absence of controlled trial data limits interpretation of these findings. Collectively, the available evidence across CNS injury models supports TNF-α as a broadly relevant therapeutic target beyond SCI and suggests that the mechanistic rationale for ETN explored in this review may extend to other acquired neurological conditions. Rigorous controlled studies are needed to confirm these findings and to determine whether the temporal and route-of-delivery considerations identified in the preclinical SCI literature are similarly applicable in TBI and related disorders.

### 4.5. Combinatorial and Synergistic Strategies

A consistent pattern across the literature was the enhanced efficacy of ETN when combined with other interventions. Co-administration with erythropoietin, methotrexate, dexamethasone, salvianolic acid B, or NSC transplantation demonstrated greater clinical improvements in post-SCI than ETN monotherapy. These combined effects likely stem from different but supportive actions, suggesting that multi-faceted approaches may provide synergistic benefits post-SCI. These findings align with the current understanding of multifactorial secondary injury processes following SCI.

### 4.6. Limitations of the Current Literature and Future Directions

Despite the promising findings seen within the included studies, several limitations warrant consideration. Animal species, injury models, and dosing and delivery regimens of ETN varied between included studies, limiting direct comparisons and generalizability of results. Notably, each of the fifteen included studies utilized a thoracic SCI model. This severely limits generalization of findings, as pathophysiology and recovery trajectories can vary significantly by injury locus—particularly between the cervical and thoracic spinal regions. Further research is necessary to determine if current findings are consistent across varying spinal levels of injury. Furthermore, there is a distinct lack of direct comparisons between ETN and other TNF inhibitors in SCI literature. One notable exception is the work of Novrup et al. (2014) [41], which compared ETN to XPro1595, a selective inhibitor of soluble TNF. In their study, both agents failed to improve functional recovery or reduce lesion size when administered systemically; however, centrally administered XPro1595, but not ETN, significantly improved functional recovery and reduced tissue damage [41]. This differential response suggests that ETN’s non-selective blockade of both soluble and transmembrane TNF may represent a mechanistic limitation in the SCI context, as transmembrane TNF signaling through TNFR-2 appears to contribute to neuroprotective effects. Nevertheless, the investigation of ETN remains scientifically justified given its well-characterized clinical safety, established dosing data across multiple conditions, and the predominantly positive results observed across most of the included studies. However, no studies in our search directly compared ETN to other clinically available TNF inhibitors, like infliximab or adalimumab.

As TNF is known to peak shortly after injury, many studies focused on short-term outcomes, which provide limited insight into chronic effects and long-term safety. No included study assessed outcomes beyond 8 weeks, and none were specifically designed to evaluate chronic neurological sequelae, long-term neuropathic pain trajectories, or sustained immunological consequences of ETN use. Whether early anti-inflammatory effects translate into durable functional recovery remains to be fully elucidated. Additionally, few studies explored dose–response relationships, pharmacokinetics, or potential systemic immunologic consequences of ETN use within SCI. These gaps must be addressed prior to the conceptualization of translational and clinical investigations.

Several directions for future research can be identified. First, standardization of dosing, therapeutic windows, and delivery modalities is needed to determine optimal timing and delivery of ETN within the treatment of SCI. Second, longitudinal studies evaluating systemic and chronic outcomes of ETN use—including potential adverse systemic effects, neuropathic pain, and neuroplasticity—are necessary to determine whether early improvements translate into meaningful benefits. Third, although ETN was compared to other therapeutics in some of the articles reviewed herein, future studies may aim to compare its efficacy with other TNF inhibitors to determine mechanism-specific benefits in SCI, as ETN’s mechanism differs from that of infliximab or adalimumab. Fourth, translational studies comparing the systemic versus central delivery of ETN, including investigations into delivery vehicles or slow-release formulations, can elucidate delivery-specific effects. Fifth, future research should prioritize comparative efficacy studies between ETN and other anti-TNF agents to better define the role of soluble versus transmembrane TNF inhibition in SCI. While the current scarcity of such studies limits the feasibility of a meta-analysis at this time, such comparisons are vital for identifying the most effective biologic intervention. Finally, given the immunologic mechanisms of ETN, safety profiling and evaluation of adverse effects after SCI—such as exacerbation of post-injury infections or immune-related complications—are essential prior to the consideration of clinical investigations.

### 4.7. Review Strengths and Limitations

This review is the first to synthesize and describe current literature regarding the therapeutic potential of ETN in SCI. By adhering to PRISMA guidelines, the current review maintains an attentive and transparent methodology. Furthermore, the use of comprehensive search strategies across different databases helped to reduce the risk of missing relevant studies. Nevertheless, this review does have its limitations. Notably, while the most recent study included in this review was published in 2021, the comprehensive search strategy used implies that this may reflect a plateau in preclinical literature. This gap in recent activity underscores the critical need for the present review; without a synthesis of the existing literature, the field lacks a clear consensus on the efficacy of etanercept and directions for future investigations. By consolidating these findings now, a necessary evidence base is established to determine whether ETN warrants further investigation. However, the lack of standardized species inclusion, injury model, and dosing and timing of treatment regimens across studies made direct comparisons challenging.

## 5. Conclusions

In conclusion, current evidence seems to support the potential of ETN as a therapeutic intervention targeted towards the modulation of the secondary injury cascade following SCI. In preclinical investigations, ETN consistently reduced proinflammatory signaling and improved early histopathological and functional outcomes. Although limitations in methodology and translational potential remain, the findings of this review provide a compelling basis for further investigation of TNF-targeted therapies in the early stages of post-SCI treatment and highlight ETN as a promising candidate for refinement and optimization.

## Figures and Tables

**Table 1:** Inclusion and Exclusion Criteria.

Included	Excluded
Original research accessible as of 9 December 2025	Pre-print articles
In vivo mammalian SCI pathology (e.g., mouse, rat, rabbit)	Case reports/series
Used etanercept as experimental or control treatment	Conference abstracts
Originally published in English language	Commentaries/Letters
	Systematic reviews
	Meta-analyses

**Table 2:** Summary of included studies.

Reference	Species			Type of SCI			Sample *n* (Total *N*)	ETN Dose	ETN Route		ETN Timing		
	Mou	Rat	Rab	Cmp	Con	H/T			Sys	Cen	Pre	Post	Del
Genovese et al., 2006 [32]	X			X			30 (120)	5 mg/kg	X		X	X	
Genovese et al., 2007 [33]	X			X			30 (240)	1.25 mg/kg	X			X	
Marchand et al., 2009 [34]		X				X	8 (32)	50 µg/day		X		X	X
Chi et al., 2010 [35]		X			X		5–10 (175)	5 mg/kg	X		X		X
Chen et al., 2011 [36]		X		X			18–45 (108)	5 mg/kg	X			X	
Ye et al., 2011 [37]		X		X			10 (90)	5 mg/kg	X			X	
Bayrakli et al., 2012 [38]			X	X			8 (24)	2.5 mg/kg	X			X	X
Vidal et al., 2013 [39]	X					X	4–15 (54)	125 µg	X				X
Wang et al., 2014 [40]		X				X	6–27 (72)	5 mg/kg	X				X
Novrup et al., 2014 [41]	X				X		3–15	10 mg/kg (Sys), 2.3 mg/mL @ 1 µL/h (Cen)	X	X		X	
Caliskan et al., 2016 [42]		X		X			5–10 (85)	1.25 mg/kg	X			X	
Gezici et al., 2017 [43]		X		X			8 (40)	1.25 mg/kg	X			X	
Hasturk et al., 2018 [44]		X		X			6 (54)	5 mg/kg	X			X	
Sun et al., 2019 [45]	X				X		6–8	10–150 µg	X				X
Shen et al., 2021 [46]	X				X		3–9	4 mg/kg	X			X	
Totals	6	8	1	8	4	3	-	-	14	2	2	10	6

SCI = spinal cord injury, ETN = etanercept, Mou = mouse, Rab = rabbit, Cmp = compression, Con = contusion, H/T = hemi- or transection, Sys = systemic, Cen = central, Pre = pre-injury, Post = immediate/within 2 h post-injury, Del = delayed/past 2 h post-injury. Sample size is reported as group n (total N) when reported. For demonstration of dose timing, studies that included multi-dose regimens are depicted according to when the first treatment was initiated.

**Table 3:** Synthesis of ETN Treatment across SCI Models and Timing.

Category	Subgroup	Examples of “Positive” Effects	Number of Studies	Positive Effect
Primary Outcomes	Inflammation	↓ TNF-α, IL-1β	12	12
	Histopathology	↑ Myelin sparing, ↓ Lesion size, ↓ Apoptosis	11	9
	Motor Function	↑ BMS/BBB/Tarlov scores	14	12
	Sensory & Pain	↓ Allodynia/Hyperalgesia	2	1
Injury Type	Compression	Any primary outcome	8	8 *
	Contusion	Any primary outcome	4	3
	Hemi-/Transection	Any primary outcome	3	2
ETN Timing	Pre-treatment	Any primary outcome	2	2
	Immediate (<2 h)	Any primary outcome	10	9
	Delayed (>2 h)	Any primary outcome	6	5

ETN = etanercept, BMS = Basso mouse scale, BBB = Basso, Beattie, and Bresnahan scale. ↓ indicates decrease, ↑ indicates increase. * One study demonstrated ETN to be effective only in combinatorial therapy, but not as monotherapy [33].

**Table 4:** Study-Level Risk of Bias Assessment Using the SYRCLE Tool.

Study	Sequence Generation	Blinded Outcome Assessment	Incomplete Outcome Data	Overall Risk
Genovese et al., 2006 [32]	Low	Low	Low	Low
Genovese et al., 2007 [33]	Low	Low	Low	Low
Marchand et al., 2009 [34]	Low	Low	Low	Low
Chi et al., 2010 [35]	Unclear	Low	Low	Moderate
Chen et al., 2011 [36]	Unclear	Unclear	Low	Moderate
Ye et al., 2011 [37]	Low	Unclear	Low	Moderate
Bayrakli et al., 2012 [38]	Unclear	Unclear	Low	Moderate
Vidal et al., 2013 [39]	Unclear	Unclear	Low	Moderate
Wang et al., 2014 [40]	Low	Low	Low	Low
Novrup et al., 2014 [41]	Low	Low	Low	Low
Caliskan et al., 2016 [42]	Unclear	Low	Low	Moderate
Gezici et al., 2017 [43]	Low	Low	Low	Low
Hasturk et al., 2018 [44]	Low	Low	Low	Low
Sun et al., 2019 [45]	Unclear	Low	Low	Moderate
Shen et al., 2021 [46]	Low	Low	Low	Low
Total	60% low risk	73% low risk	100% low risk	53% low risk

## Data Availability

No new data were created or analyzed in this study. Database search terms are available as Supplementary Materials.

## Supplementary Materials

The following supporting information can be downloaded at: https://www.mdpi.com/article/10.3390/brainsci16040388/s1. File S1: Database search queries.

## Abbreviations

The following abbreviations are used in this manuscript:

SCI	Spinal cord injury
WHO	World Health Organization
CNS	Central nervous system
ROS	Reactive oxygen species
TNF	Tumor necrosis factor
ETN	Etanercept
PRISMA	Preferred Reporting Items for Systematic reviews and Meta-Analyses
MeSH	Medical Subject Headings
