# Supplementary material for: Etanercept in Spinal Cord Injury: A Systematic Review
Source: Brain Sci. 2026 Mar 31;16(4):388. doi: 10.3390/brainsci16040388 (PMC13114609; doi:10.3390/brainsci16040388)
Supplement: Supplementary file 1 [file brainsci-16-00388-s001.zip › brainsci-4181724-supplementary.pdf]

Search model: Etanercept and spinal cord injuries

MeSH Entry Terms for "Etanercept": https://www.ncbi.nlm.nih.gov/mesh/2009879

MeSH Entry Terms for "Spinal Cord Injuries": https://www.ncbi.nlm.nih.gov/mesh/68013119

PubMed:

("Etanercept"[Mesh] OR "Etanercept"[tiab]) AND ("Spinal Cord Injuries"[Mesh] OR "Spinal Cord Injur*"[tiab])

14 August 2024: 20 results

9 December 2025: 20 results

Scopus

TITLE-ABS-KEY(("Etanercept*" OR "TNF Receptor Type II-IgG Fusion Protein*" OR "TNF Receptor Type II IgG Fusion Protein*" OR "Enbrel*" OR "TNFR-Fc Fusion Protein*" OR "Fusion Protein, TNFR-Fc*" OR "TNFR Fc Fusion Protein*" OR "TNTR-Fc*" OR "TNR 001*" OR "TNR-001*" OR "TNR001*" OR "TNT Receptor Fusion Protein*" OR "Erelzi*" OR "Etanercept-szzs*" OR "Recombinant Human Dimeric TNF Receptor Type II-IgG Fusion Protein*" OR "Recombinant Human Dimeric TNF Receptor Type II IgG Fusion Protein*") AND ("Spinal Cord Injur*"))

14 August 2024: 69 results

9 December 2025: 72 results

Web of Science

(TS=("Etanercept*" OR "TNF Receptor Type II-IgG Fusion Protein*" OR "TNF Receptor Type II IgG Fusion Protein*" OR "Enbrel*" OR "TNFR-Fc Fusion Protein*" OR "Fusion Protein, TNFR-Fc*" OR "TNFR Fc Fusion Protein*" OR "TNTR-Fc*" OR "TNR 001*" OR "TNR-001*" OR "TNR001*" OR "TNT Receptor Fusion Protein*" OR "Erelzi*" OR "Etanercept-szzs*" OR "Recombinant Human Dimeric TNF Receptor Type II-IgG Fusion Protein*" OR "Recombinant Human Dimeric TNF Receptor Type II IgG Fusion Protein*")) AND (TS=("Spinal Cord Injur*"))

14 August 2024: 27 results

9 December 2025: 27 results

Total: 119
